# Thermal products dominate FDA adverse-event reports for electrophysical agents

**DOI:** 10.3389/fmedt.2026.1993929

**Published:** 2026-10-28

**Authors:** Doğukan Kurç

**Affiliations:** Department of Physiotherapy and Rehabilitation, Faculty of Health Sciences, Istanbul Medipol University, Istanbul, Türkiye

**Keywords:** adverse events, electrophysical agents, MAUDE, medical devices, postmarket surveillance, rehabilitation, safety monitoring

## Abstract

**Introduction:**

Electrophysical agents contribute to rehabilitation and symptom management through different forms of energy. Postmarket evaluation therefore requires device-specific interpretation of reported health effects and technical problems.

**Methods:**

This retrospective descriptive study examined how technology, product-line concentration and reporting patterns shaped FDA device reports received during 2016–2025. Thirty-one product categories were searched. Device identity and application were screened, coded health effects and device problems were classified using exact-term dictionaries, and their co-occurrence was summarized by report.

**Results:**

The main analysis included 4,934 reports. Passive thermal packs and powered heating pads contributed 83.0% of the cohort. ThermaCare alone accounted for 1,705 reports (34.6%) across multiple manufacturer labels. Cutaneous codes occurred in 2,786 reports (56.5%); cutaneous and use/instruction codes co-occurred in 737. Excluding the two dominant thermal families left 840 reports, with cutaneous codes in 44.8% and neurological or neuromuscular codes in 7.9%. Electrical stimulation, optical therapy and iontophoresis showed distinct profiles. Receipt-year distributions revealed product-specific clusters, while narrative examination identified mismatches between product category and the reported operating mode. The 11 death-coded reports were characterized by the reported circumstances and attribution.

**Discussion:**

These findings support surveillance organized around identifiable product lines, actual operating modes and linked clinical–device information. Such reporting can guide targeted investigation and revision of device instructions and risk controls alongside continuing evaluation of therapeutic benefit.

## 1 Introduction

Electrophysical agents deliver electrical, electromagnetic, acoustic or thermal energy for rehabilitation and symptom management. They are used to modulate pain, activate muscle, assist movement and provide local heating or cooling. Transcutaneous electrical nerve stimulation (TENS), neuromuscular electrical stimulation (NMES), functional electrical stimulation (FES), therapeutic ultrasound and optical or thermal treatments serve different therapeutic purposes. Their selection depends on the treatment objective, the patient's condition and the properties of the energy delivered (1–3).

Therapeutic benefit is specific to the indication and treatment protocol. Johnson and colleagues' meta-TENS study found lower pain during or immediately after stimulation, and Dailey and colleagues reported improvements in movement-evoked pain and fatigue in women with fibromyalgia (4, 5). The ETRELKA trial did not support an additional benefit of TENS for symptomatic knee osteoarthritis, while Desmeules and colleagues found no added benefit of therapeutic ultrasound for rotator-cuff tendinopathy (6, 7). Reviews of low-level laser therapy and superficial heat similarly relate benefit to dose, indication or treatment period (8, 9). Appropriate selection and safe delivery are consequently part of the same therapeutic decision.

Continuing safety evaluation adds information as devices are used across patients and settings. Trials establish effects under defined protocols, whereas postmarket reports can describe component problems, unexpected exposure conditions and clinical changes during routine use. Bargeri and colleagues found heterogeneous safety reporting across reviews of physical agents, and Hunsinger and colleagues identified incomplete adverse-event reporting in nonpharmacologic pain trials (1, 10). The US Food and Drug Administration (FDA) Manufacturer and User Facility Device Experience (MAUDE) database captures suspected device problems and associated health effects through mandatory and voluntary reporting pathways (11). These reports contribute to the wider evidence used to reassess device performance, instructions and risk controls after market entry (12, 13).

Recent MAUDE analyses have also examined transcranial magnetic stimulation and robotic-assisted knee arthroplasty, illustrating the use of postmarket reports to investigate evolving medical technologies (14, 15). Previous MAUDE studies have examined dermatologic energy devices, body-contouring technologies, powered muscle stimulators and home optical products (16–19). Extending surveillance across electrophysical-agent families raises a further interpretive question: how much of a pooled profile reflects technology-specific problems, and how much reflects the products and reporting pathways that dominate the database? A registered product category, the device described and the mode used during an event may also convey different information. These distinctions matter when coded reports are used to prioritize regulatory follow-up.

This study examined health-effect and device-problem profiles across selected electrophysical-agent families. It assessed their co-occurrence, the concentration of reports within product lines, receipt-year patterns and source-supported differences between product categories and operating modes. The objective was to identify how the structure and content of postmarket reports could support more specific investigation, risk management and feedback to safe clinical use.

## 2 Materials and methods

### 2.1 Study design and data source

This retrospective descriptive study used public FDA device-event reports retrieved on September 8, 2026. The search covered 31 product categories and receipt dates from January 1, 2016 through December 31, 2025. Categories represented noninvasive electrical or magnetic stimulation, therapeutic ultrasound and combination systems, optical therapy, shortwave or microwave modalities, iontophoresis and external thermal products. Exact queries, category definitions and source specifications are provided in Supplementary Material S1. Applicable STROBE and RECORD items guided reporting (20, 21).

### 2.2 Inclusion and exclusion criteria

Eligibility was assessed from device identity, the reported application and source text. Physical-agent devices used for rehabilitation, symptom relief, muscle conditioning or other documented therapeutic applications were included. Reports of aesthetic or body-contouring applications, general wellness use and products promoted with an unverified energy mechanism distinct from the sampled physical modalities were excluded. Other exclusions covered implanted or percutaneous systems, surgery, neonatal or critical-care core-temperature management, blood/circuit warming, diagnostic-preparation warming and non-energy products. Reports submitted in error, administrative or marketing-only records without an eligible event, and a report concerning maintenance of an out-of-scope dental device were also excluded. Conventional devices with uncertain application details were retained and identified for sensitivity analysis. Residential-fire reports involving eligible heating pads were retained even when a device contribution was unconfirmed.

The searches returned 5,096 query matches representing 5,095 distinct reports. Initial product-identity screening excluded 73 records, leaving 5,022 for further screening. A further 41 exclusions yielded the broader device-code cohort of 4,981 reports. Excluding 41 aesthetic/body-contouring reports, four general-wellness reports and two reports concerning an unverified energy mechanism yielded the main cohort of 4,934 reports. The broader cohort was retained for sensitivity analysis (Supplementary Figure S1 and Table S10).

### 2.3 Classification and analysis

Technology families were assigned from the described device and energy rather than the query code alone. Optical devices were grouped together when the category did not reliably distinguish laser, light and infrared applications. A multimode device remained in its combination family; a stated event-time mode was recorded separately. A functional-neuromuscular category therefore did not by itself establish FES delivery. Conflicts between structured identity and event descriptions were retained with the supporting passages. Thermal preparation equipment was included when it heated therapeutic packs.

Detailed source-text examination covered 913 records outside the two dominant thermal families. Structured product-identity and rule-assisted narrative screening covered 4,109 records initially assigned to passive packs or powered heating pads. The rules flagged neonatal, diagnostic, surgical, circuit-warming and non-energy contexts for targeted source checking; selected application-scope decisions were retained with supporting source passages. Observed circumstances, manufacturer test findings, hypotheses and generic instructions were distinguished. Nine records underwent contextual term review, including two subsequently excluded records. Terms were removed in five included records: 13 patient-problem terms and two device-problem terms. These removals concerned explicitly denied or baseline-only conditions and terms belonging to another device. Reported worsening was retained. Clinical examples and all death-coded reports were examined separately from the quantitative code summaries. Device and application classifications and contextual code corrections were performed by the author without independent validation by a second reviewer.

The FDA report identifier was the primary unit. Duplicate retrievals and repeated terms within a report were counted once. Exact patient-problem terms were assigned to cutaneous, pain/sensory, electrical-shock, neurological/neuromuscular, musculoskeletal, ocular/auditory, cardiorespiratory, infectious, allergic, other local-tissue and other health-effect domains. Generic and missing-information codes were retained separately. Burning or itching alone did not establish a skin lesion, and general tissue-damage terms were separated from explicitly cutaneous terms. Device problems were grouped independently into thermal/fire, containment/material-integrity, electrical/output, use/instruction, contact/compatibility and other specified problems. Full mappings and contextual corrections are provided in Supplementary Material S1.

Patient-outcome indicators, including hospitalization, intervention, disability and death, were extracted independently of the event-type field. The term “death-coded reports” refers to reports with death recorded in the patient-outcome field. FDA definitions were applied to all entries in the patient-outcome array, including partially decoded entries (22–24). Multiple health domains, device problems or outcomes could occur in one report. Health–device co-occurrence required both domain flags in the same report. Use/instruction codes were treated as supplied classifications rather than adjudicated user error.

Counts and percentages used the relevant report denominator. Reporter country described the reporting field rather than patient or event location. Calendar year was assigned from FDA receipt date. Product lines were identified from normalized structured brand, generic-name and model fields, including spacing and punctuation variants, across manufacturer labels. Abbreviated or misspelled labels were resolved using the structured manufacturer and device/model information. ThermaCare was aggregated across its reporting labels. Receipt-year distributions were summarized by technology family and selected product lines. Main numerical family profiles were limited to families with at least 20 reports; all families were documented in the supplement.

Sensitivity analyses used uncorrected FDA codes, the broader device-code frame and exclusions of uncertain applications, explicit non-rehabilitation contexts, passive packs and powered heating pads, the largest manufacturer group, and the entire ThermaCare product line. Supported cross-report links were collapsed in a separate reporting-unit analysis. A further scenario collapsed identical substantial event narratives sharing the reported brand and an available matching event date. These procedures distinguished report multiplicity from patient identity (24). The analysis estimated reporting proportions and joint-code counts.

### 2.4 Ethics

The study used publicly available, de-identified regulatory reports released with FDA redactions and involved no recruitment, participant contact, intervention or attempted re-identification. No separate ethics committee review or additional participant consent was sought for this secondary analysis. Direct patient identifiers were not used or reproduced in the manuscript.

## 3 Results

Passive thermal packs and powered heating pads accounted for 83.0% of the 4,934 eligible reports. The remaining 840 reports covered stimulation, acoustic, optical and other physical-agent families (Table 1). Figure 1 shows how this technology mix contributed to the combinations of coded health effects. The reporter-country field identified the United States in 3,523 reports and was unspecified in 800; these fields did not establish where the patient received treatment.

**Table 1:** Technology families and reporting characteristics.

Characteristic	Reports, *n*	%
Electrical stimulation and multimode stimulators	413	8.4
Magnetic stimulation	5	0.1
Therapeutic ultrasound	21	0.4
Ultrasound–stimulation combination systems	88	1.8
Optical therapy	71	1.4
Shortwave or microwave modalities	5	0.1
Iontophoresis	20	0.4
Circulating hot/cold systems	196	4.0
Thermal preparation equipment	19	0.4
Powered heating pads	1,751	35.5
Passive hot/cold packs	2,343	47.5
Other mixed or uncertain systems	1	0.0
Other identified physical modalities	1	0.0
Reporting source		
User Facility	239	4.8
Manufacturer	3,826	77.5
Distributor	431	8.7
Voluntary	438	8.9
Reported event type		
Malfunction	1,670	33.8
Injury	3,214	65.1
Unspecified	28	0.6
Other	10	0.2
Death	11	0.2
No answer provided	1	0.0

Denominator: 4,934 eligible reports. Technology families describe the device/energy grouping, not a confirmed event-time mode for every report. Event types and reporting sources are retained as supplied by FDA. The 11 death event-type reports are the same records with death recorded in the patient-outcome field in Table 2.

**Figure 1:**
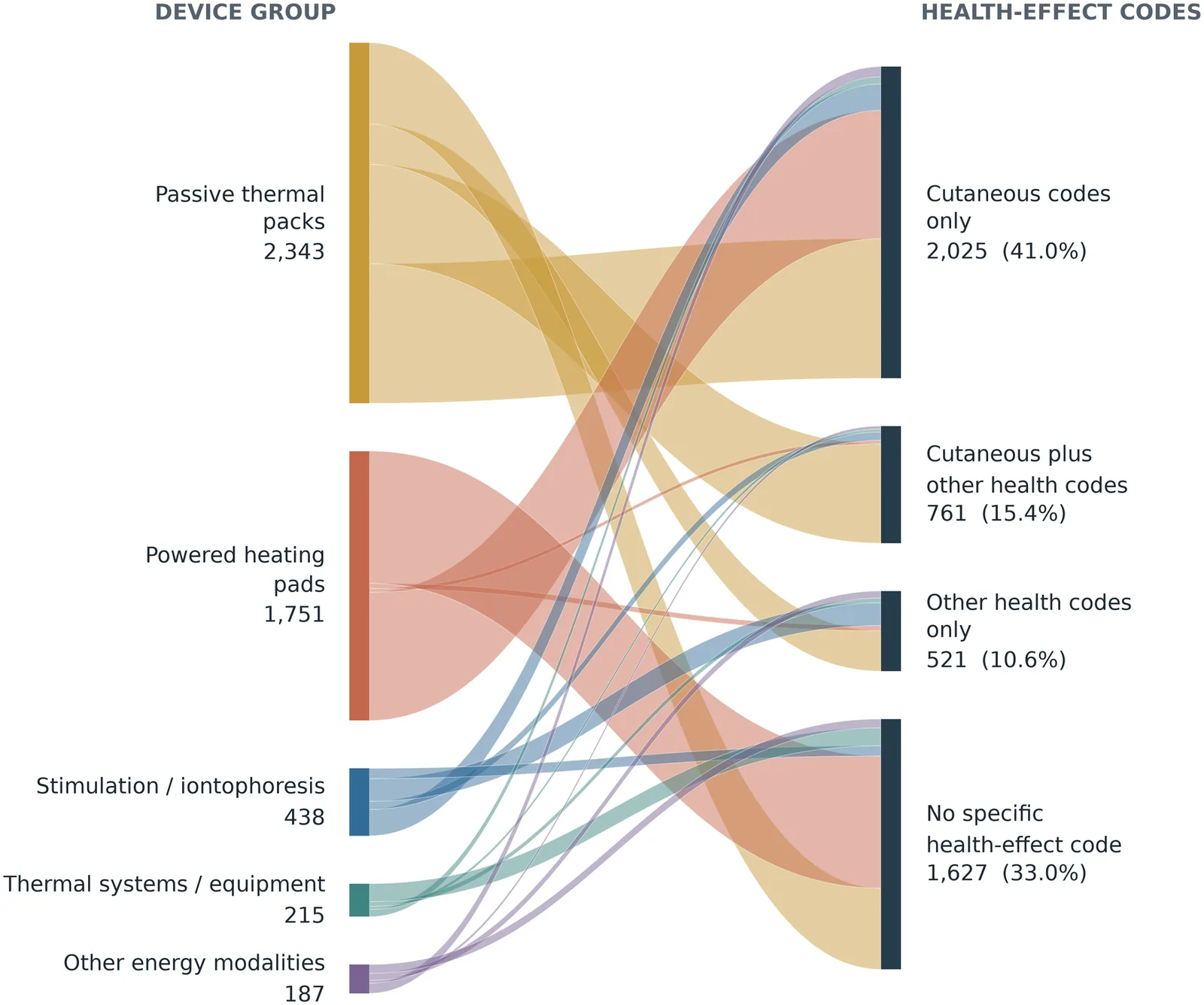
How technology mix shapes combinations of coded health effects. Each ribbon links a broad device group to one of four mutually exclusive code patterns; ribbon width represents the number of reports. Stimulation/iontophoresis includes electrical and magnetic stimulation and iontophoresis. Thermal systems/equipment includes circulating systems and pack-heating equipment. Other energy modalities include ultrasound, optical and remaining physical-agent families. No specific health-effect code is a reporting category, not confirmation of no harm. The total is 4,934 reports.

Product-line concentration extended across manufacturer labels. ThermaCare accounted for 1,705 reports (34.6%), exceeding the 1,379 reports attributed to the largest manufacturer group. Its records appeared under Pfizer, Angelini and Bridges labels (Supplementary Table S8). Excluding the entire product line left 3,229 reports, with cutaneous codes in 45.5% and use/instruction codes in 17.8% (Supplementary Table S4).

Receipt-year counts were concentrated in particular product lines (Figures 3A,B). The largest annual total occurred in 2019 (933 reports). ThermaCare contributed 539 reports in 2019. For Mighty Bliss, 241 of 322 reports were received in 2023; Thermophore contributed 192 reports in 2018. Complete family-by-year counts are provided in Supplementary Table S7. These distributions describe when reports entered the FDA dataset.

Cutaneous injury or reaction codes occurred in 2,786 reports (56.5%), while pain or abnormal sensation occurred in 652 (Table 2). The family-specific pattern extended beyond skin findings (Table 3; Figure 2). Electrical stimulation and multimode stimulators had neurological/neuromuscular codes in 46 reports (11.1%) and electrical-shock codes in 31 (7.5%). Optical devices had ocular/auditory codes in 6 reports (8.5%). Iontophoresis had cutaneous codes in 18 of 20 reports.

**Table 2:** Coded health effects, clinical consequences and device problems.

Report characteristic	Reports, *n* (%)
Cutaneous injury or reaction	2,786 (56.5%)
Pain or abnormal sensation	652 (13.2%)
Electric shock	68 (1.4%)
Neurological or neuromuscular effects	100 (2.0%)
Musculoskeletal injury or functional problem	38 (0.8%)
Ocular or auditory effects	42 (0.9%)
Cardiorespiratory or circulatory effects	44 (0.9%)
Infection	53 (1.1%)
Hypersensitivity or allergic reaction	34 (0.7%)
Other local tissue effects	477 (9.7%)
Other coded health or exposure problems	194 (3.9%)
Reported clinical consequences	
Hospitalization	138 (2.8%)
Required Intervention	1,724 (34.9%)
Disability	63 (1.3%)
Life Threatening	25 (0.5%)
Death	11 (0.2%)
Congenital Anomaly	3 (0.1%)
Other	1,445 (29.3%)
Specific health-effect code recorded	3,307 (67.0%)
Patient-outcome indicator recorded	3,065 (62.1%)
Coded device problems	
Heating, fire or combustion problems	1,049 (21.3%)
Leakage, rupture or material integrity	916 (18.6%)
Electrical, output or activation problems	343 (7.0%)
Use, instructions or human factors	967 (19.6%)
Contact, positioning or compatibility	263 (5.3%)
Other specified device problems	564 (11.4%)
Specific device-problem code recorded	3,292 (66.7%)

Denominator: 4,934 eligible reports. Health-effect groups and clinical-consequence indicators can overlap. Required intervention, hospitalization and other outcomes are regulatory report fields; they are not independent clinical adjudications. Missing indicators do not establish absence of an outcome.

**Table 3:** Selected health-effect domains within technology families with at least 20 reports.

Technology family	Cutaneous	Pain/sensation	Electric shock	Neurological	Ocular/auditory
Electrical/multimode stimulation (*n* = 413)	205 (49.6%)	83 (20.1%)	31 (7.5%)	46 (11.1%)	4 (1.0%)
Ultrasound (*n* = 21)	5 (23.8%)	3 (14.3%)	0 (0.0%)	2 (9.5%)	0 (0.0%)
Ultrasound–stimulation systems (*n* = 88)	51 (58.0%)	5 (5.7%)	10 (11.4%)	2 (2.3%)	0 (0.0%)
Optical therapy (*n* = 71)	27 (38.0%)	28 (39.4%)	0 (0.0%)	9 (12.7%)	6 (8.5%)
Iontophoresis (*n* = 20)	18 (90.0%)	2 (10.0%)	0 (0.0%)	2 (10.0%)	0 (0.0%)
Circulating hot/cold systems (*n* = 196)	66 (33.7%)	13 (6.6%)	9 (4.6%)	4 (2.0%)	0 (0.0%)
Powered heating pads (*n* = 1,751)	858 (49.0%)	20 (1.1%)	16 (0.9%)	8 (0.5%)	3 (0.2%)
Passive hot/cold packs (*n* = 2,343)	1,552 (66.2%)	495 (21.1%)	0 (0.0%)	26 (1.1%)	28 (1.2%)

Values are *n* (%), with the family-specific report denominator shown in the first column. Neurological includes neuromuscular codes. Domains overlap. All domains and smaller families are reported in Supplementary Material S1, Section S5.

The selected domains permit a compact numerical comparison; Figure 2 displays the full domain pattern for these families.

**Figure 2:**
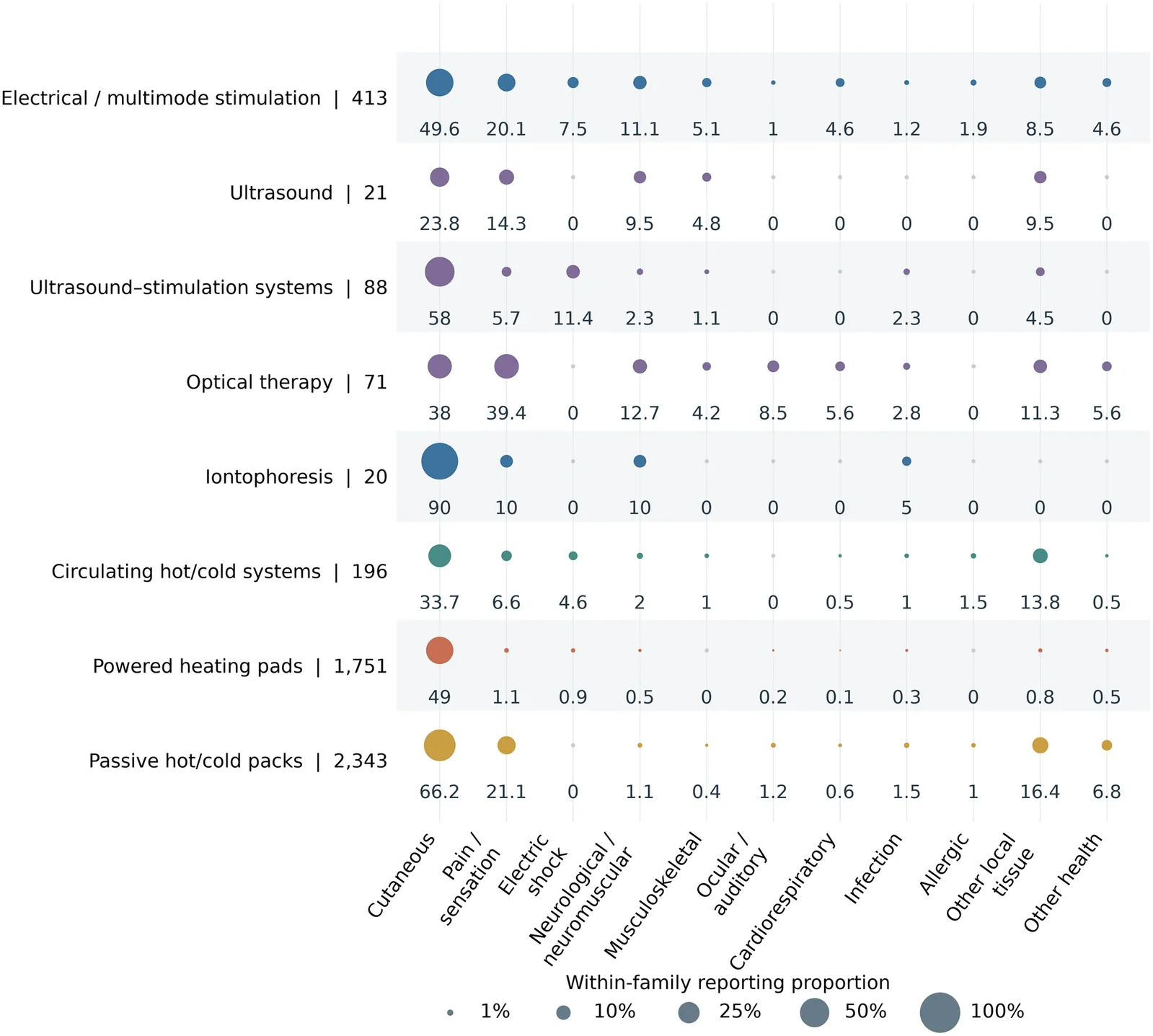
Technology-specific coded health-effect profiles for families with at least 20 reports. Circle area is proportional to the percentage of reports in each family containing the corresponding code group. Row labels give report counts. Every cell is labelled below its marker, including zero values; complete counts and smaller families appear in Supplementary Material S1, section S5. Colors identify the broad device groups used in Figure 1. Groups can overlap within a report. Percentages describe reporting composition.

**Figure 3:**
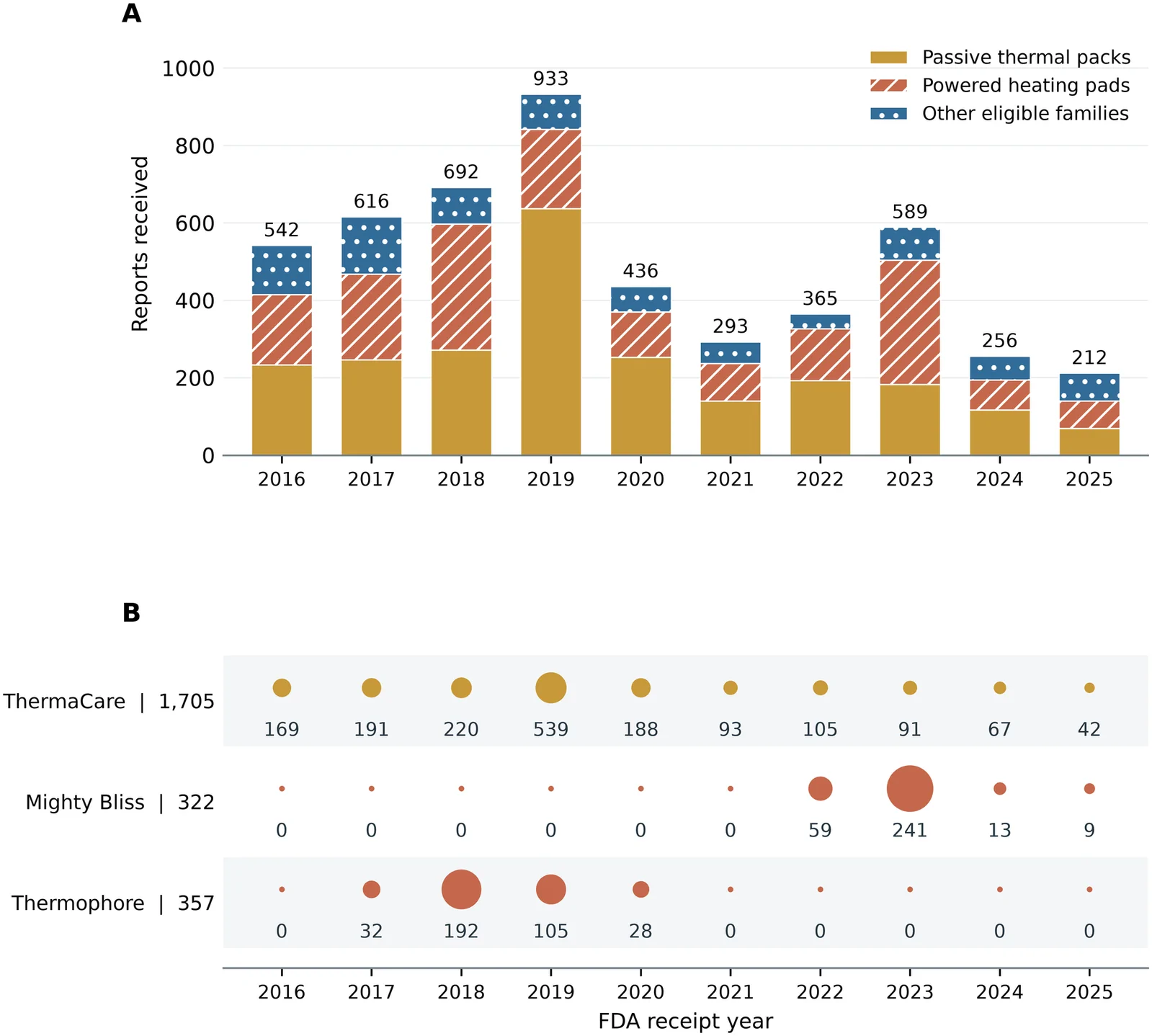
Receipt-year composition and product-line clustering. Panel **(A)** shows annual report counts for passive thermal packs, powered heating pads and other eligible families, distinguished by color and fill pattern. Labels above bars give annual totals. Panel **(B)** shows the receipt-year counts for ThermaCare, Mighty Bliss and Thermophore; circle area represents the share of each product line's reports received in that year, and every cell gives its count. The horizontal axis is the year FDA received the report. Complete family-by-year and product-line counts appear in Supplementary Tables S7 and S9.

Joint coding linked health effects to different technical problem domains (Table 4). Cutaneous and use/instruction codes co-occurred in 737 reports. After passive packs and powered heating pads were excluded, cutaneous codes co-occurred with contact/compatibility problems in 43 reports and thermal/fire problems in 41. The same restricted cohort contained 66 reports with neurological/neuromuscular codes (7.9%).

**Table 4:** Co-occurrence of health-effect and device-problem codes.

Health-effect domain	Heat/fire	Material	Output	Use	Contact	Other
Cutaneous injury or reaction	389/41	145/8	67/34	737/27	177/43	190/27
Pain or abnormal sensation	100/21	59/3	25/21	145/12	83/26	61/17
Electric shock	4/0	6/2	41/33	6/3	1/1	5/3
Neurological or neuromuscular effects	4/1	1/0	19/16	18/4	14/10	13/8
Musculoskeletal injury or functional problem	5/1	2/1	3/2	5/2	7/5	3/2
Ocular or auditory effects	2/1	24/1	3/2	4/2	6/2	4/3
Cardiorespiratory or circulatory effects	1/0	1/1	1/1	5/2	3/1	3/2
Infection	6/1	4/0	1/0	9/0	4/1	5/3
Hypersensitivity or allergic reaction	2/1	4/0	0/0	8/1	10/3	0/0
Other local tissue effects	54/4	37/6	10/6	112/9	52/15	60/9
Other coded health or exposure problems	13/1	116/0	7/5	25/0	8/2	22/6

Each cell gives the report count in the full cohort/the count after passive thermal packs and powered heating pads are excluded (denominators 4,934/840). Column groups: thermal/fire, containment/material integrity, electrical/output, use/instruction, contact/compatibility and other specified device problems. A report may contribute to several cells. Counts indicate joint coding within the same report, not causal attribution or adjudicated use error.

The 11 reports with death recorded as a patient outcome were the same 11 reports classified as death in the FDA event-type field. These death-coded reports represented different reporting circumstances. Four Sunbeam reports described fatal residential fires or associated legal/insurance allegations involving heating pads. These reports did not establish four device-caused deaths. Five ThermaCare reports mentioned a death in consumer contacts without an established product-related mechanism. One Light Relief report alleged a sequence of foot injury, infection and subsequent death. One Brain Thermal Tunnel report described hypertension, hypoxia and death after treatment in a person with amyotrophic lateral sclerosis. Two additional death-coded records in the broader device-code frame explicitly cross-referenced the same Tesla BioHealing account; these were excluded because the claimed energy mechanism was outside the defined physical-agent frame. Report-level summaries and attribution details are provided in Supplementary Material S1, Section S8.

Source examination also identified differences between category labels, reported modes and clinical circumstances. A Hill HF54 report retrieved under an ultrasound category described an electrical muscle-stimulation treatment; the multimode system was retained as a combination device. Other narratives described pack-content exposure after rupture, delayed recognition of skin changes during stimulation, and lower-limb fractures reported around FES cycling in people with osteoporosis. The FES reports differed in symptom timing and the certainty of device contribution. The supporting device identities, observations and manufacturer findings are presented in Supplementary Material S1, Section S3.

Sensitivity results showed how scope and reporting units affected the profiles (Supplementary Table S4). The broader device-code frame contained 4,981 reports. Removing the two dominant thermal families from the main cohort yielded 840 reports, with cutaneous codes in 44.8%. Supported report-linkage analysis produced 4,866 reporting units, and exact narrative/brand/date grouping produced 4,907 units. Uncertain-application, manufacturer and product-line exclusions are displayed alongside these scenarios.

## 4 Discussion

The main contribution is the identification of three features that shape electrophysical-agent surveillance: dominance by common thermal products, concentration within product lines, and differences between a registered category and the treatment actually described. Together, these features determine what a pooled safety profile represents. Family-specific summaries and joint health–device coding make the reports more useful for targeted investigation and refinement of risk controls.

The breadth of the product frame explains why this dataset differs from earlier modality-focused studies. Manudhane and colleagues found 36 MAUDE reports in their review of cleared powered muscle stimulators, whereas the present search encompassed consumer thermal products and several other device families (18). Tremaine and Avram examined dermatologic laser and energy devices, and Nguyen and colleagues separated reports from the multiple adverse events contained within body-contouring reports (16, 17). These studies establish the value of technology-specific surveillance. The present analysis adds a cross-family view in which the products contributing most reports can be identified explicitly, rather than allowing their profile to stand for every physical modality.

Product-line aggregation is necessary when a familiar product appears under changing manufacturer labels. Removing only the largest company leaves related ThermaCare records in the cohort and addresses a different question from removing the product line. Liao and colleagues describe the importance of understanding MAUDE's data structure, while Mishali and colleagues show how reporting volumes and composition change over time (25, 26). The present concentration analysis identifies a practical unit for follow-up: a consistently named product line linked to manufacturer labels, model information and report provenance. Its prominence signals where the pooled evidence comes from; clinical use and risk remain separate quantities.

Receipt-year clusters further show why surveillance counts need reporting context. The Mighty Bliss cluster was received after the FDA's published recall notice for heating pads with reported electrical, thermal and skin-injury problems (27). That chronology provides a reason to examine complaint and recall-related reporting together, without assigning the cluster a single cause. Everhart and colleagues documented delays in adverse-event reporting, which makes event-to-receipt timing a relevant variable for such an investigation (28). Follow-up should distinguish incident dates, company awareness, submission dates and recall membership before interpreting a rise or fall as a change in product performance.

The product-category mismatch illustrates a second regulatory information need: device identity and operating mode should be recorded separately. The Hill report described muscle stimulation in a system retrieved through an ultrasound category. A category-level summary alone would place that event within a different treatment context. Machal's function-oriented MAUDE analysis similarly connects device functions and reported problems to risk-management questions (29). For multimode rehabilitation devices, the corresponding record should identify the selected mode, accessories, settings and sequence of use. This would support assessment of the relevant function while preserving the identity of the complete device.

Joint health–device coding can focus investigation on the treatment interface. Among cutaneous reports outside the dominant thermal families, the largest joint-code counts involved contact/compatibility and thermal problems, directing attention to electrodes, coupling materials and contact conditions. Zugerman described dermatitis and burns associated with electrode materials, and Dittmar and colleagues documented electrode-associated sensitization; these are distinct clinical processes despite a shared application site (30, 31). Kuhn and colleagues' electrode work and the stimulation analyses of Merrill and colleagues and Cogan and colleagues explain why geometry and electrical delivery matter (32–34). A post-event generator test should therefore be interpreted alongside the components and application conditions actually examined.

Family-specific profiles also identify outcomes that skin-focused monitoring would miss. Hill and colleagues identified ocular injury in 28 of 87 reports concerning home optical devices, supporting explicit attention to eye exposure in optical-device follow-up (19). Their device selection differs from the broader optical family examined here, so the proportions describe different report sets. FDA advice on circulating hot/cold systems instead emphasizes barriers, exposure duration and observation of treated tissue (35). These examples support technology-specific instructions and complaint forms that capture the affected person, exposure pathway and evolving symptoms.

The FES cycling narratives show why clinical vulnerability remains part of a device investigation. Lai and colleagues reported partial attenuation of distal-femur bone loss, whereas Eser and colleagues found no significant attenuation of tibial cortical bone loss; Gorgey and colleagues further examined site- and protocol-specific musculoskeletal effects of NMES and FES (36–38). The fracture reports therefore belong within an assessment of loading, skeletal fragility and the intended therapeutic objective. Established precautions and the spinal-cord-injury bone-health guideline support assessment of bone status, movement restrictions and impaired sensation (2, 39). Recording new swelling, treatment sequence and clinical assessment would make subsequent review more informative than a device name and a fracture code alone.

Death-coded records require the same separation of report status from clinical attribution. The consumer contacts, residential-fire allegations and injury-sequence allegation represented different evidentiary situations. Their source content should guide the follow-up requested: clinical course and cause of death, identification of the product involved, contemporaneous investigation and the basis for attributing a contribution. Ensign and Cohen's discussion of report structure and multiplicity is especially relevant when linked records describe one account (24). Explicit characterization also prevents unverified-energy products retrieved through a familiar code from silently entering a clinical modality denominator.

The study has limitations. MAUDE contains selected, incomplete and sometimes contradictory reports, without device-use denominators or independent causal verification (11, 25). Receipt-year patterns are influenced by reporting delay and source practices; the retrieval date also defines the available view of the final study years. The selected categories do not encompass every physical agent, and some conventional-device applications remain uncertain. Narrative examination was more detailed outside the dominant thermal families, and coded outcomes were not clinically adjudicated. Device/application classifications and contextual code corrections were made by a single reviewer without independent validation, leaving the possibility of classification error. Explicit scope rules, separate product-line analyses, complete family summaries and reporting-unit sensitivity analyses preserve the descriptive contribution while defining its interpretation.

### 4.1 Recommendations for surveillance and future research

The regulatory value of these findings lies in connecting surveillance to specific follow-up actions. Bralić and colleagues position real-world and postmarket information within continuing evidence generation, while Kramer and colleagues describe complementary surveillance approaches across regulatory systems (12, 13). Here, product-line naming would improve aggregation, mode and component fields would improve investigation, and linked clinical information would improve interpretation of coded outcomes. A prospective, modality-specific registry could then connect treatment exposure and patient vulnerability to observed outcomes. It could evaluate whether revised instructions, interface checks or symptom-monitoring procedures improve safe delivery while preserving the intended therapeutic contribution.

## 5 Conclusion

Thermal products and product-line concentration shape the pooled FDA reporting profile for electrophysical agents. Technology-specific summaries reveal additional health-effect patterns, and joint health–device coding identifies more focused questions for investigation. Surveillance that links consistent product identity, actual operating mode, application conditions and clinical follow-up can support continuing refinement of device instructions and risk controls alongside evidence of therapeutic benefit.

## Data Availability

Publicly available datasets were analyzed in this study. This data can be found here: FDA Manufacturer and User Facility Device Experience (MAUDE), accessed through the openFDA Device Adverse Event Reports dataset: https://api.fda.gov/device/event.json. Documentation: https://open.fda.gov/apis/device/event/. The 31 product-code searches, FDA receipt-date interval (January 1, 2016–December 31, 2025), retrieval date (September 8, 2026) and coding definitions are provided in Supplementary Material S1. Derived analytic records, source-linked review tables and analysis scripts are available from the corresponding author on reasonable request.
