# Supplementary material for: Thermal products dominate FDA adverse-event reports for electrophysical agents
Source: Front Med Technol. 2026 Oct 28;8:1993929. doi: 10.3389/fmedt.2026.1993929 (PMC13752072; doi:10.3389/fmedt.2026.1993929)
Supplement: Supplementary file 1 [file Datasheet1.docx]

Supplementary Material S1

Thermal products dominate FDA adverse-event reports for electrophysical agents

S1. Retrieval, selection and measurement

The fixed retrieval date was September 8, 2026. Product-code searches selected records by date received from January 1, 2016 through December 31, 2025. Each query used the openFDA device/event endpoint with a product-code condition and the receipt-date interval. Results were downloaded in complete pages and reconciled against the reported query total. Source responses and hashes were retained. A report appearing under more than one queried category was counted once by its FDA report identifier. Table S1 lists the search frame, and Figure S1 shows record selection.

The analysis was retrospective and exploratory, with no prospective registration. The final analytic protocol and all changes were retained internally. Quantitative outcomes were obtained from coded fields. Technology and application screening combined detailed source-text examination of 913 records outside the two dominant thermal families with structured product-identity and rule-assisted narrative screening of 4,109 thermal-family records. Rules flagged neonatal, diagnostic, surgical, circuit-warming and non-energy contexts; selected classifications were checked against retained source passages. Device/application classifications and contextual code corrections were performed by the author without independent second-reviewer validation. Screening does not imply that every thermal narrative was read in full. Narrative examples were kept separate from the coded outcome counts.

**Table S1. Product-category search frame**

| **FDA code** | **Category description** | **Query matches** |
| --- | --- | --- |
| GZJ | Transcutaneous nerve stimulator for pain relief | 201 |
| NUH | Over-the-counter transcutaneous nerve stimulator | 92 |
| IPF | Stimulator, Muscle, Powered | 66 |
| NGX | Stimulator, Muscle, Powered, For Muscle Conditioning | 86 |
| GZI | External functional neuromuscular stimulator | 16 |
| LIH | Interferential current therapy | 6 |
| IMI | Ultrasonic Diathermy For Use In Applying Therapeutic Deep Heat | 28 |
| PFW | Stationary Ultrasonic Diathermy Device For Use In Applying Therapeutic Deep Heat | 0 |
| LXF | Diathermy, Ultrasonic, For Use Other Than Applying Therapeutic Deep Heat | 0 |
| IMG | Stimulator, Ultrasound And Muscle, For Use In Applying Therapeutic Deep Heat | 82 |
| NHN | Powered Light Based Laser Non-Thermal Instrument With Non-Heating Effect For Adjunctive Use In Pain Therapy | 3 |
| NZY | Light, Lymphedema Reduction, Low Energy | 0 |
| IOB | Lamp, Infrared, Non Heating | 0 |
| ILY | Lamp, Infrared, Therapeutic Heating | 85 |
| IMJ | Diathermy, Shortwave, For Use In Applying Therapeutic Deep Heat | 3 |
| ILX | Diathermy, Shortwave, For Use Other Than Applying Therapeutic Deep Heat | 3 |
| IOA | Diathermy, Microwave, For Use In Applying Therapeutic Deep Heat | 0 |
| HPH | Diathermy, Microwave, For Use Other Than Applying Therapeutic Deep Heat | 0 |
| IRT | Pad, Heating, Powered | 1749 |
| IRQ | Unit, Heating, Powered | 21 |
| IMA | Pack, Heat, Moist | 8 |
| IME | Pack, Hot Or Cold, Reusable | 111 |
| IMD | Pack, Hot Or Cold, Disposable | 2295 |
| ILO | Pack, Hot Or Cold, Water Circulating | 218 |
| LBG | Device, Fluidized Therapy, Dry Heat | 0 |
| EGJ | Device, Iontophoresis, Other Uses | 23 |
| KTB | Device, Iontophoresis, Specific Uses | 0 |
| NYY | Stimulator, Muscle, Powered, With Limited Output, For Rehabilitation | 0 |
| NYZ | Stimulator, Muscle, Powered, With Limited Output, For Muscle Conditioning | 0 |
| SDO | Transcutaneous Electrical Spine Stimulator To Improve Skeletal Muscle Strength And Sensation | 0 |
| SGT | Electromagnetic Stimulator For Healthy Muscle Stimulation | 0 |

A zero count means no matching report was returned by this query in the specified interval. It is not a safety conclusion or evidence that the modality was unused.

The 5,096 query matches represented 5,095 distinct reports. Initial product-identity screening excluded 73 records. Of the 5,022 records screened in further detail (913 outside the two dominant thermal families and 4,109 thermal-family records), 41 were excluded, leaving the broader cohort of 4,981. A further 47 were excluded: 41 aesthetic/body-contouring reports, four general-wellness reports and two Tesla BioHealing reports with an unverified energy mechanism. These 47 records are listed in Table S10. The final cohort comprised 4,934 reports, including eight with uncertain application details; the latter were removed in a sensitivity analysis. Figure S1 shows the stages and exclusion categories.

S2. Clinical and device term dictionaries

The following lists reproduce the decoded FDA terms used for classification. Within-report repeated terms were deduplicated. Terms and groups may overlap; report-level percentages must not be added. General tissue/scarring terms were kept distinct from explicitly cutaneous terms. An absent code was not converted into a confirmed negative clinical finding.

Cutaneous injury or reaction

Burn(s); Partial thickness (Second Degree) Burn; Burn, Thermal; Superficial (First Degree) Burn; Full thickness (Third Degree) Burn; Caustic/Chemical Burns; Lead(s), Burn(s) From; Radiation Burn; Cryogenic Burn; Erythema; Blister; Rash; Skin Irritation; Skin Discoloration; Skin Inflammation/ Irritation; Peeling; Urticaria; Skin Tears; Skin Inflammation; Skin Erosion; Pressure Sore/Ulcer; Localized Skin Lesion; Wheal(s); Contact Dermatitis; Skin Disorders; Eczema; Skin Infection.

Pain or abnormal sensation

Pain; Discomfort; Burning Sensation; Itching Sensation; Skin Burning Sensation; Tingling; Numbness; Headache; Chest Pain; Abdominal Pain; Neck Pain; Eye Pain; Arthralgia; Limb Pain; Myalgia; Sensitivity of Teeth; Ear Pain; Back pain; Pelvic Pain; Hyperesthesia; Hypoesthesia; Increased Sensitivity; Hyperresponsive to stimuli; Hyporesponsive to stimuli.

Electric shock

Electric Shock; Shock from Patient Lead(s); Electrocution.

Neurological or neuromuscular effects

Tingling; Numbness; Undesired Nerve Stimulation; Nerve Damage; Dizziness; Cramp(s); Muscle Spasm(s); Neuropathy; Paralysis; Shaking/Tremors; Loss of consciousness; Device Overstimulation of Tissue; Convulsion/Seizure; Cramp(s) /Muscle Spasm(s); Brain Injury; Muscular Rigidity; Seizures; Muscle Weakness/Atrophy; Seizures, Grand-Mal; Transient Ischemic Attack; Convulsion, Tonic; Twitching; Hemorrhage, Cerebral; Hyperresponsive to stimuli; Hyporesponsive to stimuli; Hyperesthesia; Epilepsy; Hypoesthesia; Tics/Tremor; Vertigo.

Musculoskeletal injury or functional problem

Ambulation or Postural Difficulties; Fall; Bone Fracture(s); Hernia; Loss of Range of Motion; Muscle/Tendon Damage; Unspecified Musculoskeletal problem; Neck Stiffness; Rheumatoid Arthritis; Limited Mobility Of The Implanted Joint; Joint Swelling; Limb Fracture; Intervertebral Disc Compression or Protrusion; Joint effusion; Muscle Weakness/Atrophy.

Ocular or auditory effects

Red Eye(s); Eye Injury; Blurred Vision; Eye Burn; Tinnitus; Foreign Body Sensation in Eye; Dry Eye(s); Eye Pain; Visual Impairment; Keratitis; Loss of Vision; Retinal Detachment; Hearing Loss; Vitreous Floaters; Corneal Abrasion; Unspecified Eye / Vision Problem; Acoustic Shock; Eye Irritation; Ear Pain; Vitreous Hemorrhage.

Cardiorespiratory or circulatory effects

Dyspnea; High Blood Pressure/ Hypertension; Tachycardia; Arrhythmia; Palpitations; Thrombosis; Atrial Fibrillation; Low Blood Pressure/ Hypotension; Cardiac Arrest; Unspecified Respiratory Problem; Increased Respiratory Rate; Hypovolemic Shock; Respiratory Failure; Respiratory Acidosis; Heart Failure; Atrial Flutter; Myocardial Infarction; Respiratory Distress; Non specific EKG/ECG Changes; Electro-Mechanical Dissociation; Vascular System (Circulation), Impaired; Unspecified Heart Problem; Hypoxia.

Infection

Unspecified Infection; Purulent Discharge; Skin Infection; Wound Infection; Bacterial Infection; Cellulitis; Abscess; Viral Infection; Post Operative Wound Infection; Fungal Infection.

Hypersensitivity or allergic reaction

Hypersensitivity/Allergic reaction.

Other local tissue effects

Swelling; Scarring; Scar Tissue; Tissue Damage; Hemorrhage/Blood Loss/Bleeding; Inflammation; Bruise/Contusion; Irritation; Ulcer; Abrasion; Rupture; Fluid Discharge; Reaction; Tissue Breakdown; Necrosis; Swelling/ Edema; Local Reaction; Discharge; Impaired Healing; Unspecified Tissue Injury; Contusion; Laceration(s); Blood Loss; Edema; Erosion; Deformity/ Disfigurement; Hyperemia; Ecchymosis; Necrosis Of Flap Tissue; Wound Dehiscence; Gangrene; Hematoma; Adhesion(s); Swollen Lymph Nodes/Glands; Nodule; Abnormal scarring.

Other coded health or exposure problems

Abdominal Cramps; Abdominal Distention; Alteration In Body Temperature; Alteration in Body Temperature; Anxiety; Aspiration/Inhalation; Chemical Exposure; Chills; Cognitive Changes; Collapse; Confusion/ Disorientation; Cyst(s); Dehydration; Dementia; Depression; Device Embedded In Tissue or Plaque; Diaphoresis; Diarrhea; Dysuria; Emotional Changes; Exposure to Body Fluids; Failure of Implant; Fatigue; Fever; Gastritis; Heartburn/Indigestion; Heavier Menses; Hot Flashes/Flushes; Hyperglycemia; Hypoglycemia; Incomplete Induced Abortion; Irritability; Malaise; Menstrual Irregularities; Nausea; Radiation Exposure, Unintended; Shock; Sleep Dysfunction; Sore Throat; Sweating; Unspecified Gastrointestinal Problem; Vomiting.

Generic and information-status terms

No effect: No Known Impact Or Consequence To Patient; No Clinical Signs, Symptoms or Conditions; No Consequences Or Impact To Patient.

Missing: Insufficient Information; No Information; No Code Available; Appropriate Clinical Signs, Symptoms and Conditions Term/Code Not Available; Not Applicable.

Generic: Injury; Patient Problem/Medical Problem; Complaint, Ill-Defined; Disability; Therapeutic Effects, Unexpected; Inadequate Pain Relief; Death; Muscle Stimulation.

No Patient Involvement was retained separately. Burn codes: Burn(s); Partial thickness (Second Degree) Burn; Burn, Thermal; Superficial (First Degree) Burn; Full thickness (Third Degree) Burn; Caustic/Chemical Burns; Lead(s), Burn(s) From; Radiation Burn; Cryogenic Burn.

Heating, fire or combustion problems

Fire; Excessive Heating; Overheating of Device; Temperature Problem; Thermal Decomposition of Device; Smoking; Melted; Insufficient Heating; Flare or Flash; Charred.

Leakage, rupture or material integrity

Leak/Splash; Burst Container or Vessel; Break; Fluid/Blood Leak; Material Rupture; Unintended Ejection; Material Split, Cut or Torn; Explosion; Material Twisted/Bent; Bent; Crack; Material Deformation; Material Integrity Problem; Material Protrusion/Extrusion; Cut In Material; Hole In Material; Material Puncture/Hole; Material Separation; Torn Material; Structural Problem; Gel Leak; Split; Fracture; Material Disintegration; Chemical Spillage; Material Frayed; Material Fragmentation; Failure to Seal; Peeled/Delaminated; Degraded; Material Discolored; Scratched Material; Corroded.

Electrical, output or activation problems

Sparking; Therapeutic or Diagnostic Output Failure; Electrical /Electronic Property Problem; Inappropriate/Inadequate Shock/Stimulation; Failure to Shut Off; Output Problem; Electrical Shorting; Unintended Electrical Shock; Intermittent Energy Output; Energy Output Problem; Self-Activation or Keying; Loss of Power; Failure to Power Up; Output above Specifications; High impedance; Power Conditioning Problem; Device Stops Intermittently; Activation Problem; Unintended Power Up; Electronic Property Issue; Failure to Conduct; Circuit Failure; Unexpected Shutdown; Electromagnetic Interference; Delayed Charge Time; Failure to Fire; Improper Device Output; Electrical Overstress; Arcing; Power Problem; Device Remains Activated; Electrical Power Problem; Activation Failure; Battery Problem; Charging Problem; Failure to Charge; Premature Activation; Device Inoperable; Communication or Transmission Problem; Application Program Problem.

Use, instructions or human factors

Improper or Incorrect Procedure or Method; Use of Device Problem; Labelling, Instructions for Use or Training Problem; Off-Label Use; Device Markings/Labelling Problem; Human Factors Issue; Device Handling Problem; Human-Device Interface Problem; Inadequate User Interface; Device Difficult to Setup or Prepare; Use of Incorrect Control/Treatment Settings; Inadequate or Insufficient Training; Lack of Maintenance Documentation or Guidelines; Missing Information; Expiration Date Error; Device Expiration Issue.

Contact, positioning or compatibility

Patient-Device Incompatibility; Patient Device Interaction Problem; Loss of or Failure to Bond; Device Slipped; Loose or Intermittent Connection; Failure To Adhere Or Bond; Inadequacy of Device Shape and/or Size; Positioning Problem; Detachment of Device or Device Component; Fitting Problem; Detachment Of Device Component; Connection Problem; Device Dislodged or Dislocated; Activation, Positioning or Separation Problem; Device-Device Incompatibility; Compatibility Problem; Component Incompatible; Migration or Expulsion of Device; Unintended Compatibility; Positioning Failure; Unintended Movement.

Other specified device problems

Component Missing; Contamination; Contamination /Decontamination Problem; Defective Component; Defective Device; Deformation Due to Compressive Stress; Device Alarm System; Device Contamination with Chemical or Other Material; Device Damaged Prior to Receipt by User; Device Damaged Prior to Use; Device Displays Incorrect Message; Device Emits Odor; Device Operates Differently Than Expected; Device Operational Issue; Device Packaging Compromised; Device Unsafe to Use in Environment; Difficult or Delayed Separation; Difficult to Open or Close; Difficult to Remove; Entrapment of Device; Expulsion; Fail-Safe Design Failure; Fail-Safe Did Not Operate; Fail-Safe Problem; Failure to Advance; Failure to Auto Stop; Failure to Clean Adequately; Failure to Cut; Folded; Improper Flow or Infusion; Incomplete or Missing Packaging; Incorrect, Inadequate or Imprecise Result or Readings; Increase in Pressure; Invalid Sensing; Key or Button Unresponsive/not Working; Lack of Effect; Low Test Results; Manufacturing, Packaging or Shipping Problem; Material Too Rigid or Stiff; Mechanical Problem; Mechanics Altered; Microbial Contamination of Device; Moisture Damage; Moisture or Humidity Problem; Naturally Worn; No Display/Image; No Pressure; Noise, Audible; Nonstandard Device; Out-Of-Box Failure; Packaging Problem; Physical Property Issue; Physical Resistance/Sticking; Product Quality Problem; Protective Measures Problem; Short Fill; Sticking; Suction Problem; Tear, Rip or Hole in Device Packaging; Unexpected Therapeutic Results; Unsealed Device Packaging.

Generic/missing device terms: Adverse Event Without Identified Device or Use Problem; Insufficient Device Problem Information; Appropriate Device Problem Term/Code Not Available; Device Issue; No Apparent Adverse Event; null.

S3. Clinical-consequence indicators and narrative examples

FDA patient-outcome entries were parsed as report-level sets. Both expanded labels and residual letter codes were decoded: D, death; L, life threatening; H, hospitalization; S, disability; C, congenital anomaly; R, required intervention; O, other; U, unknown; I, no information; A, not applicable; *, invalid data. Sequence-number artifacts were discarded. The report was the unit even when patient arrays or outcome strings contained multiple entries. These indicators were not inferred from the separate event-type field. All 11 included reports carrying a death patient-outcome code were also classified as death in the event-type field; the two sets contained the same report identifiers.

Official code source: FDA MDR Data Files, patient-file outcome field. https://www.fda.gov/medical-devices/medical-device-reporting-mdr-how-report-medical-device-problems/mdr-data-files (accessed September 8, 2026).

C1. Interferential stimulation

The report described tap water as a conductive medium, no electrode-retaining straps and an electric heating pad over clothing and electrodes. Burns were recognized after treatment, without reported discomfort during application. The returned generator was reported to operate within specification. This did not reproduce all original contact conditions.

Interpretation: Reported application circumstances and later generator testing; no independently established injury mechanism. The two records concern one linked reporting episode.

FDA report 6440604: https://www.accessdata.fda.gov/scripts/cdrh/cfdocs/cfmaude/detail.cfm?mdrfoi__id=6440604

FDA report 6440647: https://www.accessdata.fda.gov/scripts/cdrh/cfdocs/cfmaude/detail.cfm?mdrfoi__id=6440647

C2. Chemical cold pack

An attorney's report described a cold pack leaking through its container during use for elbow/forearm pain, followed by reported burns and subsequent treatment. The narrative describes alleged injury and treatment; it does not establish an independent causal investigation.

Interpretation: Reported leakage and clinical consequences; attorney attribution is not independent verification of causation or later diagnoses.

FDA report 7128107: https://www.accessdata.fda.gov/scripts/cdrh/cfdocs/cfmaude/detail.cfm?mdrfoi__id=7128107

C3. FES cycling

Two reports described lower-limb fractures in wheelchair users with osteoporosis. One described acute knee pain during cycling followed by imaging-detected fractures; the other described ankle swelling noticed the next day in a person without lower-limb sensation. Device contribution was uncertain in the second report because the injury time was unknown. The manufacturer reported no device malfunction.

Interpretation: Two distinct fracture reports, not an incidence estimate or proof that FES caused both fractures. They illustrate existing patient-selection considerations.

FDA report 15815080: https://www.accessdata.fda.gov/scripts/cdrh/cfdocs/cfmaude/detail.cfm?mdrfoi__id=15815080

FDA report 16203668: https://www.accessdata.fda.gov/scripts/cdrh/cfdocs/cfmaude/detail.cfm?mdrfoi__id=16203668

C4. Laser system

A report described unintended laser activation during lens replacement, with the beam striking a technician's eye. Required safety eyewear was reportedly not worn. The report identifies the handling task and exposure circumstance; permanent visual injury was not established by this description.

Interpretation: Reported occupational eye exposure and missing eyewear; lasting visual loss was not established in the source description.

FDA report 22396327: https://www.accessdata.fda.gov/scripts/cdrh/cfdocs/cfmaude/detail.cfm?mdrfoi__id=22396327

C5. Magnetic stimulation

A staff member's pacemaker was replaced after abnormalities were detected around the period of magnetic-device use in another room. The pacemaker already had corroded leads and was under monitoring. The manufacturer could not exclude interference but did not establish a direct causal relationship with the hospitalization and surgery.

Interpretation: Potential interaction with a strong alternative explanation; neither a causal event nor a safe-distance estimate can be inferred.

FDA report 9380230: https://www.accessdata.fda.gov/scripts/cdrh/cfdocs/cfmaude/detail.cfm?mdrfoi__id=9380230

C6. Hill HF54 combination system

The report was retrieved under the ultrasonic-diathermy category, while the event narrative explicitly described an electrical muscle-stimulation treatment. Discomfort under the pads and subsequent blisters were reported. Manufacturer documentation describes an ultrasound platform with interferential and premodulated-current channels (3). The system was classified as an ultrasound–stimulation combination; electrical stimulation was recorded as the reported event mode.

Interpretation: The event narrative establishes a reported electrical-stimulation application, but does not specify the exact waveform, delivered intensity or injury mechanism.

FDA report 13506406: https://www.accessdata.fda.gov/scripts/cdrh/cfdocs/cfmaude/detail.cfm?mdrfoi__id=13506406

S4. Sensitivity analyses

Table S4 compares selected reporting proportions under the specified inclusion, coding and reporting-unit scenarios.

**Table S4. Selected outcomes under alternative reporting-unit and inclusion scenarios**

| **Analysis** | **Units, n** | **Cutaneous codes** | **Neurological codes** | **Use/instruction codes** |
| --- | --- | --- | --- | --- |
| Main analysis | 4934 | 2,786 (56.5%) | 100 (2.0%) | 967 (19.6%) |
| Broader device-code frame before scope restriction | 4981 | 2,806 (56.3%) | 104 (2.1%) | 974 (19.6%) |
| Uncorrected FDA-coded fields | 4934 | 2,786 (56.5%) | 102 (2.1%) | 967 (19.6%) |
| Uncertain records excluded | 4926 | 2,785 (56.5%) | 100 (2.0%) | 967 (19.6%) |
| Passive packs and powered heating pads excluded | 840 | 376 (44.8%) | 66 (7.9%) | 53 (6.3%) |
| Largest manufacturer group excluded | 3555 | 1,766 (49.7%) | 77 (2.2%) | 613 (17.2%) |
| Explicit non-rehabilitation contexts excluded | 4649 | 2,608 (56.1%) | 86 (1.8%) | 931 (20.0%) |
| Supported report families collapsed | 4866 | 2,743 (56.4%) | 97 (2.0%) | 961 (19.7%) |
| Exact narrative, brand and date groups collapsed | 4907 | 2,778 (56.6%) | 100 (2.0%) | 965 (19.7%) |
| ThermaCare product line excluded | 3229 | 1,470 (45.5%) | 76 (2.4%) | 576 (17.8%) |

Values are n (%). Linkage scenarios count reporting units, not confirmed unique patients. Exact narrative matching required a substantial event description, the same reported brand and an available matching event date. Manufacturer boilerplate alone was not treated as a confirmed duplicate episode.

S5. Technology-specific coded health effects

Electrical stimulation and multimode stimulators (n=413)

Cutaneous injury or reaction: 205 (49.6%); Pain or abnormal sensation: 83 (20.1%); Electric shock: 31 (7.5%); Neurological or neuromuscular effects: 46 (11.1%); Musculoskeletal injury or functional problem: 21 (5.1%); Ocular or auditory effects: 4 (1.0%); Cardiorespiratory or circulatory effects: 19 (4.6%); Infection: 5 (1.2%); Hypersensitivity or allergic reaction: 8 (1.9%); Other local tissue effects: 35 (8.5%); Other coded health or exposure problems: 19 (4.6%).

Magnetic stimulation (n=5)

Cutaneous injury or reaction: 1 (20.0%); Pain or abnormal sensation: 0 (0.0%); Electric shock: 1 (20.0%); Neurological or neuromuscular effects: 0 (0.0%); Musculoskeletal injury or functional problem: 0 (0.0%); Ocular or auditory effects: 0 (0.0%); Cardiorespiratory or circulatory effects: 2 (40.0%); Infection: 0 (0.0%); Hypersensitivity or allergic reaction: 0 (0.0%); Other local tissue effects: 1 (20.0%); Other coded health or exposure problems: 0 (0.0%).

Therapeutic ultrasound (n=21)

Cutaneous injury or reaction: 5 (23.8%); Pain or abnormal sensation: 3 (14.3%); Electric shock: 0 (0.0%); Neurological or neuromuscular effects: 2 (9.5%); Musculoskeletal injury or functional problem: 1 (4.8%); Ocular or auditory effects: 0 (0.0%); Cardiorespiratory or circulatory effects: 0 (0.0%); Infection: 0 (0.0%); Hypersensitivity or allergic reaction: 0 (0.0%); Other local tissue effects: 2 (9.5%); Other coded health or exposure problems: 0 (0.0%).

Ultrasound–stimulation combination systems (n=88)

Cutaneous injury or reaction: 51 (58.0%); Pain or abnormal sensation: 5 (5.7%); Electric shock: 10 (11.4%); Neurological or neuromuscular effects: 2 (2.3%); Musculoskeletal injury or functional problem: 1 (1.1%); Ocular or auditory effects: 0 (0.0%); Cardiorespiratory or circulatory effects: 0 (0.0%); Infection: 2 (2.3%); Hypersensitivity or allergic reaction: 0 (0.0%); Other local tissue effects: 4 (4.5%); Other coded health or exposure problems: 0 (0.0%).

Optical therapy (n=71)

Cutaneous injury or reaction: 27 (38.0%); Pain or abnormal sensation: 28 (39.4%); Electric shock: 0 (0.0%); Neurological or neuromuscular effects: 9 (12.7%); Musculoskeletal injury or functional problem: 3 (4.2%); Ocular or auditory effects: 6 (8.5%); Cardiorespiratory or circulatory effects: 4 (5.6%); Infection: 2 (2.8%); Hypersensitivity or allergic reaction: 0 (0.0%); Other local tissue effects: 8 (11.3%); Other coded health or exposure problems: 4 (5.6%).

Shortwave or microwave modalities (n=5)

Cutaneous injury or reaction: 1 (20.0%); Pain or abnormal sensation: 2 (40.0%); Electric shock: 0 (0.0%); Neurological or neuromuscular effects: 1 (20.0%); Musculoskeletal injury or functional problem: 0 (0.0%); Ocular or auditory effects: 1 (20.0%); Cardiorespiratory or circulatory effects: 1 (20.0%); Infection: 0 (0.0%); Hypersensitivity or allergic reaction: 0 (0.0%); Other local tissue effects: 0 (0.0%); Other coded health or exposure problems: 1 (20.0%).

Iontophoresis (n=20)

Cutaneous injury or reaction: 18 (90.0%); Pain or abnormal sensation: 2 (10.0%); Electric shock: 0 (0.0%); Neurological or neuromuscular effects: 2 (10.0%); Musculoskeletal injury or functional problem: 0 (0.0%); Ocular or auditory effects: 0 (0.0%); Cardiorespiratory or circulatory effects: 0 (0.0%); Infection: 1 (5.0%); Hypersensitivity or allergic reaction: 0 (0.0%); Other local tissue effects: 0 (0.0%); Other coded health or exposure problems: 0 (0.0%).

Circulating hot/cold systems (n=196)

Cutaneous injury or reaction: 66 (33.7%); Pain or abnormal sensation: 13 (6.6%); Electric shock: 9 (4.6%); Neurological or neuromuscular effects: 4 (2.0%); Musculoskeletal injury or functional problem: 2 (1.0%); Ocular or auditory effects: 0 (0.0%); Cardiorespiratory or circulatory effects: 1 (0.5%); Infection: 2 (1.0%); Hypersensitivity or allergic reaction: 3 (1.5%); Other local tissue effects: 27 (13.8%); Other coded health or exposure problems: 1 (0.5%).

Thermal preparation equipment (n=19)

Cutaneous injury or reaction: 1 (5.3%); Pain or abnormal sensation: 0 (0.0%); Electric shock: 1 (5.3%); Neurological or neuromuscular effects: 0 (0.0%); Musculoskeletal injury or functional problem: 0 (0.0%); Ocular or auditory effects: 0 (0.0%); Cardiorespiratory or circulatory effects: 0 (0.0%); Infection: 0 (0.0%); Hypersensitivity or allergic reaction: 0 (0.0%); Other local tissue effects: 0 (0.0%); Other coded health or exposure problems: 0 (0.0%).

Powered heating pads (n=1,751)

Cutaneous injury or reaction: 858 (49.0%); Pain or abnormal sensation: 20 (1.1%); Electric shock: 16 (0.9%); Neurological or neuromuscular effects: 8 (0.5%); Musculoskeletal injury or functional problem: 0 (0.0%); Ocular or auditory effects: 3 (0.2%); Cardiorespiratory or circulatory effects: 1 (0.1%); Infection: 6 (0.3%); Hypersensitivity or allergic reaction: 0 (0.0%); Other local tissue effects: 14 (0.8%); Other coded health or exposure problems: 9 (0.5%).

Passive hot/cold packs (n=2,343)

Cutaneous injury or reaction: 1,552 (66.2%); Pain or abnormal sensation: 495 (21.1%); Electric shock: 0 (0.0%); Neurological or neuromuscular effects: 26 (1.1%); Musculoskeletal injury or functional problem: 10 (0.4%); Ocular or auditory effects: 28 (1.2%); Cardiorespiratory or circulatory effects: 15 (0.6%); Infection: 35 (1.5%); Hypersensitivity or allergic reaction: 23 (1.0%); Other local tissue effects: 385 (16.4%); Other coded health or exposure problems: 160 (6.8%).

Other mixed or uncertain systems (n=1)

Cutaneous injury or reaction: 0 (0.0%); Pain or abnormal sensation: 0 (0.0%); Electric shock: 0 (0.0%); Neurological or neuromuscular effects: 0 (0.0%); Musculoskeletal injury or functional problem: 0 (0.0%); Ocular or auditory effects: 0 (0.0%); Cardiorespiratory or circulatory effects: 1 (100.0%); Infection: 0 (0.0%); Hypersensitivity or allergic reaction: 0 (0.0%); Other local tissue effects: 0 (0.0%); Other coded health or exposure problems: 0 (0.0%).

Other identified physical modalities (n=1)

Cutaneous injury or reaction: 1 (100.0%); Pain or abnormal sensation: 1 (100.0%); Electric shock: 0 (0.0%); Neurological or neuromuscular effects: 0 (0.0%); Musculoskeletal injury or functional problem: 0 (0.0%); Ocular or auditory effects: 0 (0.0%); Cardiorespiratory or circulatory effects: 0 (0.0%); Infection: 0 (0.0%); Hypersensitivity or allergic reaction: 0 (0.0%); Other local tissue effects: 1 (100.0%); Other coded health or exposure problems: 0 (0.0%).

Families with at least 20 reports are displayed in Figure 2. Every displayed cell shows its percentage, using one decimal place where needed; smaller families are documented here with their counts. Clinical groups may overlap. Device-family membership is not a measure of treatment exposure.

S6. Source-confirmed contextual code corrections

The following corrections concern semantic ownership or timing, not causal adjudication. Nine records underwent contextual term review, including two subsequently excluded Tesla BioHealing records. Terms were removed in five included reports. These author-performed decisions were not independently validated. Raw FDA terms remain in the retained source records and the uncorrected-code sensitivity. Domain flags were recomputed from corrected term sets. Urticaria and contact dermatitis were retained as cutaneous codes but were not assumed to demonstrate allergy without the explicit hypersensitivity/allergic-reaction term. Muscle Stimulation alone was treated as context rather than an adverse neurological effect.

Report 7259132: Neuropathy and rheumatoid arthritis occurred in an explicit list of conditions denied in the source narrative. Both terms were removed from the analytic patient-problem set.

Patient terms removed: Neuropathy; Rheumatoid Arthritis.

Report 9682315: Rheumatoid arthritis, neuropathy and impaired circulation were explicitly denied in the narrative. These terms were removed; the separately reported burn, blister and pain terms were retained.

Patient terms removed: Neuropathy; Rheumatoid Arthritis; Vascular System (Circulation), Impaired.

Report 18374964: The source described cognitive symptoms, epilepsy, fatigue, malaise, tinnitus and viral illness before stimulation. An earlier seizure-like episode was unconfirmed and predated treatment. These terms were removed. Worsened sleep dysfunction, dizziness and muscle cramps were retained as reported after treatment.

Patient terms removed: Cognitive Changes; Convulsion/Seizure; Epilepsy; Fatigue; Malaise; Tinnitus; Viral Infection.

Report 18607177: The implant-failure term referred to an earlier implant history, while the current report described a separate external TENS lead or wire problem. The patient term was removed from the analytic outcome set.

Patient terms removed: Failure of Implant.

Report 9380230: Corrosion and failure to conduct referred to pre-existing pacemaker leads rather than the sampled magnetic stimulator. These device terms were removed. Electromagnetic interference was retained as a reported possibility with uncertain attribution.

Device terms removed: Corroded; Failure to Conduct.

S7. Receipt-year and product-line distributions

Calendar year was assigned from the date FDA received the report. The distributions describe report receipt, including delayed or clustered submissions. They are not event-year incidence curves. Product lines were identified from normalized structured device identity fields, including spacing and punctuation variants, across manufacturer labels. Abbreviated or misspelled Mighty Bliss labels were resolved from the manufacturer and heating-pad identity/model fields in 12 reports. A narrative-only mention was insufficient for assignment.

**Table S7. FDA receipt year by technology family**

| **Technology family** | **2016** | **2017** | **2018** | **2019** | **2020** | **2021** | **2022** | **2023** | **2024** | **2025** |
| --- | --- | --- | --- | --- | --- | --- | --- | --- | --- | --- |
| Electrical / multimode stimulation | 73 | 84 | 48 | 28 | 30 | 16 | 16 | 50 | 33 | 35 |
| Magnetic stimulation | 0 | 0 | 0 | 1 | 1 | 0 | 0 | 1 | 1 | 1 |
| Ultrasound | 1 | 4 | 0 | 5 | 6 | 3 | 0 | 0 | 1 | 1 |
| Ultrasound–stimulation systems | 7 | 4 | 0 | 15 | 11 | 14 | 15 | 7 | 5 | 10 |
| Optical therapy | 2 | 9 | 1 | 6 | 5 | 4 | 1 | 15 | 14 | 14 |
| Shortwave / microwave | 0 | 2 | 0 | 1 | 0 | 0 | 0 | 1 | 0 | 1 |
| Iontophoresis | 3 | 3 | 8 | 2 | 2 | 0 | 0 | 0 | 0 | 2 |
| Circulating hot/cold systems | 40 | 42 | 37 | 27 | 7 | 14 | 5 | 11 | 7 | 6 |
| Thermal preparation equipment | 1 | 0 | 0 | 6 | 4 | 5 | 1 | 0 | 1 | 1 |
| Powered heating pads | 182 | 222 | 325 | 205 | 117 | 97 | 134 | 321 | 77 | 71 |
| Passive hot/cold packs | 233 | 246 | 272 | 637 | 253 | 140 | 193 | 183 | 117 | 69 |
| Mixed / uncertain systems | 0 | 0 | 0 | 0 | 0 | 0 | 0 | 0 | 0 | 1 |
| Other physical modalities | 0 | 0 | 1 | 0 | 0 | 0 | 0 | 0 | 0 | 0 |
| All eligible reports | 542 | 616 | 692 | 933 | 436 | 293 | 365 | 589 | 256 | 212 |

All entries are report counts. Annual columns sum to the corresponding total in the final row.

**Table S8. Product-line counts across reporting manufacturer labels**

| **Product line** | **Manufacturer label or consolidated group** | **Reports, n** |
| --- | --- | --- |
| ThermaCare | Pfizer | 1346 |
| ThermaCare | Angelini | 195 |
| ThermaCare | BRIDGES CONSUMER HEALTHCARE | 159 |
| ThermaCare | PF Consumer Healthcare | 4 |
| ThermaCare | UNSPECIFIED | 1 |
| Mighty Bliss | Bellavie/Sunbright | 247 |
| Mighty Bliss | Whele | 70 |
| Mighty Bliss | Ningbo Bella Vie Electricals | 2 |
| Mighty Bliss | UNSPECIFIED | 1 |
| Mighty Bliss | INTERTEK /WHELE LLC. | 1 |
| Mighty Bliss | UNKNOWN | 1 |
| Thermophore | Battle Creek Equipment | 357 |

Spacing, punctuation and clearly attributable company-name variants were consolidated within each product line. Original labels are retained in the analytic records. Manufacturer labels identify the report source and do not establish product ownership at the event date.

**Table S9. Receipt-year distribution of selected product lines**

| **Product line** | **2016** | **2017** | **2018** | **2019** | **2020** | **2021** | **2022** | **2023** | **2024** | **2025** |
| --- | --- | --- | --- | --- | --- | --- | --- | --- | --- | --- |
| ThermaCare | 169 | 191 | 220 | 539 | 188 | 93 | 105 | 91 | 67 | 42 |
| Mighty Bliss | 0 | 0 | 0 | 0 | 0 | 0 | 59 | 241 | 13 | 9 |
| Thermophore | 0 | 32 | 192 | 105 | 28 | 0 | 0 | 0 | 0 | 0 |

These report counts underlie Figure 3B. Product-line totals are identified from structured device fields.

S8. Reports carrying a death outcome

In the main cohort, the 11 reports with a death patient outcome were the same 11 reports with death recorded as the FDA event type. The four Sunbeam residential-fire reports were retained because they involved heating pads within the eligible device frame; eligibility did not require confirmation of device causation.

Sunbeam heating pads: residential-fire reports (n=4 reports). Four included reports concerned fatal residential fires. Three conveyed insurer or attorney allegations involving a heating pad. In report 6087477, pads were found during the investigation but were not identified as the fire cause; oxygen use and smoking were also described. These reports do not establish four device-caused deaths.

FDA report identifiers: 6087477, 6526794, 8615857, 9672210.

ThermaCare: deaths mentioned in consumer contacts (n=5 reports). Five included reports mentioned a death in consumer or follow-up contacts. The narratives did not establish a product-related fatal mechanism. Contexts included prior product use, an expiration-date inquiry and a later notification of death. Report 9257382 also described diabetes and elevated blood glucose. The presence of the regulatory death code was preserved separately from attribution.

FDA report identifiers: 8495511, 8495737, 9257382, 9263822, 13509734.

Light Relief: alleged injury-to-death sequence (n=1 reports). One included report alleged a foot injury after infrared-device use, subsequent infection and edema, and later death. Medication and other clinical circumstances were also described. The narrative was retained as an allegation of a clinical sequence rather than a verified fatal mechanism.

FDA report identifiers: 6085455.

Brain Thermal Tunnel: deterioration after treatment (n=1 reports). One included report described a person with amyotrophic lateral sclerosis who reportedly developed hypertension and hypoxia after treatment, was hospitalized and died days later. The source identified a thermal device but did not provide the treatment settings or establish the causal contribution. A reference to another death in the narrative was not counted as a separate report.

FDA report identifiers: 23888122.

Tesla BioHealing: excluded cross-referenced reports (n=2 reports). Two excluded reports explicitly cross-referenced the same consumer account involving pre-existing serious illness, lack of the expected improvement and a later death. They were retained in the broader device-code sensitivity only. The products were excluded from the main physical-agent cohort because their claimed energy mechanism was not an established modality within the sampled frame. These two report identifiers do not represent evidence of two independent patients.

FDA report identifiers: 21304922, 21304929.

S9. Application-scope exclusions

Reports explicitly describing aesthetic/body-contouring or general wellness applications were excluded. Two cross-referenced Tesla BioHealing reports were excluded because the claimed energy mechanism was distinct from the sampled physical modalities. Their infrared-lamp category did not establish a therapeutic heating device. Brain Thermal Tunnel was retained as a thermal system, and the Hill HF54 was classified as an ultrasound–stimulation combination system with a separately documented electrical-stimulation event mode.

The FDA warning letter documents the absence of a heating element in the Tesla products and their use claims outside the infrared-lamp category (2). The Hill platform configuration is documented by its manufacturer (3).

**Table S10. Source-supported exclusions of aesthetic or wellness applications**

| **FDA report** | **Device identity** | **Exclusion basis** |
| --- | --- | --- |
| 6294201 | IN LIGHT WELLNESS SYSTEM | General wellness application |
| 6385633 | IN LIGHT WELLNESS SYSTEMS LED LIGHT THERAPY DEVICE | General wellness application |
| 6945929 | ACCENT XL | Aesthetic/body-contouring application |
| 8404853 | ACCENT | Aesthetic/body-contouring application |
| 8744437 | OPATRA SYNERGY \| OPATRA SYNERGY NECK | Aesthetic/body-contouring application |
| 8847189 | EMSCULPT | Aesthetic/body-contouring application |
| 8857086 | TRISTAR ONE- PHOTONS SYSTEM | Aesthetic/body-contouring application |
| 9803064 | COOLTONE APPLICATOR | Aesthetic/body-contouring application |
| 9881201 | COOLTONE | Aesthetic/body-contouring application |
| 9954560 | COOLTONE | Aesthetic/body-contouring application |
| 10096340 | HEALY | General wellness application |
| 10513807 | EMSCULPT | Aesthetic/body-contouring application |
| 11189654 | COOLTONE | Aesthetic/body-contouring application |
| 11243741 | BEMER PRO-SET | General wellness application |
| 11596296 | INFRARED &LYMPHATIC DRAINAGE BODY WRAP | Aesthetic/body-contouring application |
| 12584735 | COOLTONE APPLICATOR | Aesthetic/body-contouring application |
| 12925074 | EMSCULPT \| EMSCULPT \| EMSCULPT \| EMSCULPT \| EMSCULPT | Aesthetic/body-contouring application |
| 15268853 | UNK COOLTONE \| UNK COOLTONE | Aesthetic/body-contouring application |
| 15547919 | COOLTONE APPLICATOR | Aesthetic/body-contouring application |
| 15563090 | PLATINUMLED THERAPY LIGHTS: BIOMAX 900 | Aesthetic/body-contouring application |
| 15612396 | COOLTONE APPLICATOR | Aesthetic/body-contouring application |
| 15784247 | COOLTONE SYSTEM | Aesthetic/body-contouring application |
| 16630181 | EVOLVE X INMODE | Aesthetic/body-contouring application |
| 16665462 | COOLTONE SYSTEM | Aesthetic/body-contouring application |
| 16690421 | COOLTONE SYSTEM | Aesthetic/body-contouring application |
| 17218742 | EMSCULPT NEO | Aesthetic/body-contouring application |
| 17692651 | COOLTONE SYSTEM | Aesthetic/body-contouring application |
| 17804948 | TRILIFT | Aesthetic/body-contouring application |
| 18402847 | COOLTONE | Aesthetic/body-contouring application |
| 19209963 | LIMELIGHT | Aesthetic/body-contouring application |
| 19652586 | LIMELIGHT | Aesthetic/body-contouring application |
| 19660718 | LIMELIGHT | Aesthetic/body-contouring application |
| 20558452 | BLUE LIGHT THERAPY FOR SKIN ON MY FACE | Aesthetic/body-contouring application |
| 21304922 | TESLA MEDBED GENERATOR | Unverified energy mechanism distinct from the sampled physical modalities (Tesla BioHealing) |
| 21304929 | TESLA MEDBED GENERATOR | Unverified energy mechanism distinct from the sampled physical modalities (Tesla BioHealing) |
| 21662846 | COOLTONE | Aesthetic/body-contouring application |
| 22024215 | EVOLVE SYSTEM WITH TRANSFORM APPLICATOR | Aesthetic/body-contouring application |
| 22025722 | EVOLVE SYSTEM WITH TRANSFORM APPLICATOR | Aesthetic/body-contouring application |
| 22152974 | COOLTONE SYSTEM | Aesthetic/body-contouring application |
| 22185098 | COOLTONE SYSTEM | Aesthetic/body-contouring application |
| 23022863 | COOLTONE SYSTEM | Aesthetic/body-contouring application |
| 23286628 | COOLTONE SYSTEM | Aesthetic/body-contouring application |
| 23378729 | COOLTONE | Aesthetic/body-contouring application |
| 23486787 | COOLTONE | Aesthetic/body-contouring application |
| 23845754 | COOLTONE SYSTEM | Aesthetic/body-contouring application |
| 23864498 | COOLTONE | Aesthetic/body-contouring application |
| 23907449 | EMSCULPT NEO | Aesthetic/body-contouring application |

Figure S1. Selection of FDA electrophysical-agent reports received during 2016–2025. One duplicate query match was removed. Initial product-identity screening excluded 73 records; subsequent assessment excluded 41; application-scope exclusions removed a further 47. The 4,981-report broader cohort was retained for sensitivity analysis. The final cohort contained 4,934 reports, including eight with uncertain application details.

References

1. U.S. Food and Drug Administration. MDR Data Files. Available online at: https://www.fda.gov/medical-devices/medical-device-reporting-mdr-how-report-medical-device-problems/mdr-data-files (accessed September 8, 2026).

2. U.S. Food and Drug Administration. Tesla BioHealing, Inc. - Warning Letter 658010. Available online at: https://www.fda.gov/inspections-compliance-enforcement-and-criminal-investigations/warning-letters/tesla-biohealing-inc-658010-08102023 (accessed September 9, 2026).

3. Hill Laboratories. Thin-Style Gel Pads for Ultrasound: HF54 product configuration. Available online at: https://hilllabs.com/product/thin-style-gel-pads-box-of-10/ (accessed September 9, 2026).
